# The Relationship Between Accessibility to Food Destinations and Places for Physical Activity and Children’s BMI: A Sex-Stratified Analysis

**DOI:** 10.3390/nu18030493

**Published:** 2026-02-02

**Authors:** Javier Molina-García, Xavier Delclòs-Alió, Isaac Estevan, Ana Queralt

**Affiliations:** 1 AFIPS Research Group, Department of Teaching of Physical Education, Arts and Music, University of Valencia, Avda. dels Tarongers, 4, 46022 Valencia, Spain; isaac.estevan@uv.es; 2 Epidemiology and Environmental Health Joint Research Unit, FISABIO-UJI-UV, Avda, de Catalunya, 21, 46020 Valencia, Spain; ana.queralt@uv.es; 3 Grup de Recerca en Anàlisi Territorial i Estudis Turístics (GRATET), Department de Geografia, Universitat Rovira i Virgili, Carrer Joanot Martorell, 15, 43480 Vila-seca, Spain; xavier.delclos@urv.cat; 4 AFIPS Research Group, Department of Nursing, University of Valencia, 46010 Valencia, Spain

**Keywords:** obesity, built environment, food environment, fast-food restaurant, walkability, socio-economic status, exercise, environment design

## Abstract

**Background/Objectives**: Few studies have simultaneously evaluated spatial accessibility to both food destinations and spaces for physical activity in relation to body weight in children. The aim of this study was to determine whether spatial accessibility to food destinations and places for physical activity is associated with body weight among children, differentiating between boys and girls. Neighborhood socio-economic status and walkability were incorporated as covariates. **Methods**: This cross-sectional study was conducted in Valencia, Spain. The initial sample comprised a sample of 808 children. GIS-based procedures were used to evaluate access to food outlets, walkability and socio-economic status (i.e., household income) among home neighborhoods. Access to different types of food destinations and destinations where children can engage in physical activity was assessed using the Neighborhood Environment Walkability Scale for Youth (NEWS-Y-IPEN). Weight and height were objectively assessed. The body mass index (BMI) percentile was calculated. Mixed-model regression analyses were performed. **Results**: Being a boy was positively associated with BMI percentile (*p* = 0.003), whereas physical activity was negatively related to this outcome (*p* = 0.028) in the whole sample. None of the built environment attributes were significantly associated with BMI percentile in boys. Access to healthy restaurants (*p* = 0.035), as well as neighborhood income (*p* = 0.049), were negatively associated with BMI percentile among girls. **Conclusions**: The relationship between built environmental attributes and BMI varies significantly between boys and girls. Understanding these differences is key for designing effective public health interventions with the aim of reducing childhood obesity.

## 1. Introduction

Excess body weight in childhood is strongly associated with the risk of obesity in adulthood and with an elevated risk of non-communicable diseases such as cardiovascular disease, diabetes, and some types of cancer [1,2]. Research indicates that one in five children or adolescents experience excess weight, although these figures vary depending on the region and socio-economic status (SES) [3]. In countries such as Spain, the prevalence of obesity is 18.6%, and overweight is 13.5% among children aged 1–14 years [4]. Considering these figures, childhood obesity and overweight are a serious public health problem worldwide. Identifying predictors of childhood body weight therefore remains a priority in public health research.

According to the literature, the neighborhood in which young people live plays a key role in influencing opportunities for healthy eating and for being physically active [5,6,7,8,9]. In relation to food environmental influences, greater access to unhealthy food destinations (e.g., fast food) has been associated, in some studies, with an unhealthy body weight in children, while greater access to healthier food destinations (e.g., supermarkets) has been related with a healthier body weight [9,10]. The presence of parks and playgrounds in the neighborhood, as well as the accessibility to sports or recreation facilities, have been associated with active lifestyles and healthier body weight in children [11,12,13]. Furthermore, evidence suggests that the relation between neighborhood characteristics and children’s obesity may differ by sex [9,10,14]. For example, it is likely that boys and girls vary not only in how much they are exposed to certain environmental attributes but also in how sensitive they are to these elements or how they behave in response to them. This is especially relevant when analyzing children’s lifestyle, since girls have lower levels of physical activity and are more constrained in independent mobility (outdoor) compared to boys [15,16,17]. Studies on the influence of built environment attributes on physical activity and obesity, taking sex differences into account, are very limited [14]. In the case of the relationship between access to food destinations and obesity in children, some studies indicate that there are clear sex differences in how access to food destinations impacts obesity (e.g., [9,10]). For instance, it seems that girls are more influenced by the availability of healthy and unhealthy food outlets such as supermarkets and greengrocers. That is why sex-segregated research is needed.

Neighborhood walkability (i.e., the ease with which residents can safely and conveniently reach everyday destinations on foot) and SES can modify the strength of built environmental associations with health behaviors and weight [18,19,20]. In low-walkability and low-SES areas, limited access to healthy food, fewer safe play facilities, and less well maintained and less attractive areas may intensify environmental risks.

Few studies have simultaneously evaluated spatial accessibility to both food destinations and spaces for physical activity in relation to children’s body weight. It is also necessary to conduct studies that disaggregate the results for boys and girls, incorporating neighborhood walkability and SES into a unified analytic model. The paucity of sex-sensitive environmental research in childhood obesity has been noted as a gap in the research agenda [21]. Addressing this gap is critical for designing effective urban planning and public health interventions. In this context, the aim of the present study was to determine whether spatial accessibility to food destinations and places for physical activity is related to body weight among children, differentiating between boys and girls. Neighborhood SES and walkability characteristics of the neighborhoods were also considered in the analysis.

## 2. Materials and Methods

### 2.1. Study Design and Participants

**Figure 1:**
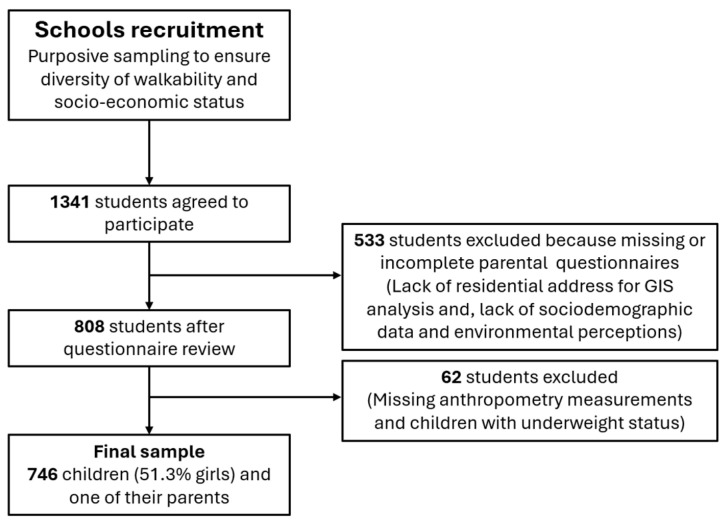
Flowchart of study sample selection process.

The data used in this cross-sectional study were collected between 2021 and 2023 as part of the Built Environment and Active Children (BEACH) project conducted in Valencia and its metropolitan area (see for more details [22]). The BEACH study followed the methodology of the International Physical Activity and the Environment Network (IPEN) in Spain [23]. Participants were recruited from ten non-randomly selected primary schools, chosen to represent a wide range of objectively assessed neighborhood walkability and SES, determined using geographic information system (GIS) procedures (see for more details [22]). Each school selected participating class groups. Eligible participants were children aged 6–12 years who lived in the study area and were able to walk independently. Of the 1341 students who initially agreed to participate, 533 were excluded, mainly because parental questionnaires—containing socio-demographic data and perceptions of the built environment—were missing or incomplete. In the case of the present study, 62 children were excluded because they did not have weight and height measurements or were underweight. A direct person-level comparison between included and excluded children was not feasible, as the missing questionnaires contained the primary exposure data (residential location and perceived environmental data). Therefore, the specific neighborhood characteristics of the non-responders remain unknown. However, the potential for selection bias is mitigated by our sampling design. As previously indicated, schools were selected based on their census-block-level SES and walkability. The final analyzed sample demonstrates a balanced distribution across the four environmental strata defined by the study design. Specifically, the sample is distributed as follows: 28.1% in High SES/High Walkability, 22.7% in High SES/Low Walkability, 25.7% in Low SES/High Walkability, and 23.6% in Low SES/Low Walkability. This balanced representation across all four quadrants ensures that the study captures a wide range of environmental exposures, mitigating the risk that the final results are driven by a single neighborhood type. The final sample consisted of 746 children (51.3% girls; mean age = 10.07 years; SD = 1.05) and one of their parents (*n* = 746 parents; 78% women; mean age = 42.67 years; SD = 5.47). Figure 1 shows the flow diagram of the study sample selection process.

### 2.2. Measures

Body mass index (BMI, kg/m^2^) was calculated as the dependent variable. Weight and height were directly measured using standardized protocols by using a stadiometer scale (SECA, Hamburg, Germany) and a bioelectrical impedance scale (Tanita BC-601, Tokyo, Japan). The Centers for Disease Control and Prevention (CDC) growth charts [24] were used to calculate BMI percentile (adjusted for age and sex) and weight-status category (i.e., “overweight”, BMI in the 85th to 95th percentile; and “obesity”, BMI ≥ 95th percentile).

Parents completed a paper version of the Neighborhood Environment Walkability Scale for Youth (NEWS-Y-IPEN) [25]. The NEWSY-IPEN has good construct and factorial validity [25]. To minimize self-referential bias, the scale uses specific, descriptive items rather than general evaluative questions, thereby reducing the influence of the participant’s current mood or general physical activity levels on their environmental ratings. This instrument has eight subscales (e.g., land use mix, recreational facilities, safety from crime, and aesthetics). In the present study, in relation to the items on land use mix, items related to food destinations and places for physical activity were selected: convenience/corner store/small grocery store; supermarket; fast-food restaurant; non-fast-food restaurant; coffee place; recreation/exercise facility (public/private); small public park; and large public park. Parents reported how long it would take them to walk to these destinations from their home. Response options were as follows: 1–5 min (5); 6–10 min (4); 11–20 min (3); 21–30 min (2); 31+ min (1); don’t know (1). A public park proximity score was calculated by averaging responses for small public park and large public park [25]. In the present study, the internal consistency (Cronbach’s alpha) for the public park indicator was 0.64. In addition, a healthy restaurant proximity index was calculated as the difference between the accessibility to fast-food restaurants and the accessibility score to non-fast-food restaurants (scale 1–5, with 5 indicating the closest proximity). This approach follows previous research that summarizes the composition of the food environment using relative indices (e.g., retail food environment index) and studies that employ proximity-based measures to examine associations with health outcomes [26,27]. To ensure this composite construct was robust and not a source of bias, we conducted preliminary sensitivity analyses by examining the independent associations of fast-food and non-fast-food accessibility with BMI percentile. Both components showed consistent and logical directions of association before being integrated, confirming that the composite measure effectively captures the relative healthfulness of the local restaurant landscape rather than being driven by a single variable.

In addition to the self-reported destinations mentioned, we included an indicator of the presence of fresh food in the neighborhood as well as a walkability measure. These were calculated using the Global Healthy and Sustainable City Indicators (GHSCI) open-source software [28,29], which provides standardized procedures for assessing urban health environments based on data from OpenStreetMap (OSM). GHSCI software (v4.10.0) includes a measure of access to fresh food markets as well as a local walkability index computed at a 100 m grid resolution. Walkability is measured based on population density, intersection density, and a composite score of access to daily destinations. Based on participants’ residential locations, both access and walkability indices were assigned individually. Neighborhood SES was assessed using the *Atlas de distribución de renta de los hogares* from Spain’s Instituto Nacional de Estadística [30]. This registry-based statistical operation provides annual estimates of average net income per person at the census tract level across Spain. For each participant, the mean per-person income of their census tract of residence was assigned as a continuous variable.

Other aspects related to the overall walkability of the neighborhood were evaluated, such as safety from crime and neighborhood aesthetics. These two neighborhood attributes were assessed using the NEWS-Y-IPEN questionnaire. Safety from crime is a scale constructed by averaging 4 items regarding the fear of a child being hurt by a stranger in different situations (i.e., when alone outside around the home, when with a friend outside around the home, when walking alone or with a friend in local streets, when alone or with a friend in local park), whereas aesthetics is a scale based on 3 items (interesting things, beautiful/natural things, building/home nice to look at). Responses were provided on a 4-point scale, from strongly disagree (1) to strongly agree (4). Higher scores indicated higher perceived safety. In the present study, the internal consistency (Cronbach’s alpha) for the perceived subscales was α = 0.96 for safety from crime and α = 0.77 for neighborhood aesthetics, indicating good reliability.

By integrating both perceived measures (via NEWS-Y-IPEN) and objective GIS-based data (via GHSCI/OSM), we aimed to capture the multi-dimensional nature of the built environment. This approach acknowledges that physical availability (objective measures) and functional accessibility or safety (perceptions) represent distinct, non-redundant dimensions that provide complementary information, as they often capture different pathways through which the environment influences health behaviors.

Parents also reported the date of birth and sex of their child. Moreover, they indicated their own sex and age and home postal address. Finally, physical activity was self-reported by children using the Spanish version of the Physical Activity Questionnaire for Children (PAQ-C; [31]). This questionnaire records physical activity over the past 7 days and consists of 10 sections. Previous research has indicated that section nine is the most valid for assessing physical activity in children [32]. Specifically, section nine asks children how often they did physical activity each day of the week (such as sports, playing, dancing, or any other physical activity). All days (from Monday to Sunday) are rated on a 5-point Likert scale from 1 (low) to 5 (high). Scores from this section were averaged to yield a score between 1 and 5.

### 2.3. Data Analysis

Data were analyzed using SPSS v.28.0 (SPSS, Chicago, IL, USA). Descriptive statistics (e.g., means, standard deviations, frequencies, and percentages) were calculated. Mixed linear regression analyses (SPSS MIXED procedure) assessed the relationship of each independent variable (i.e., food destinations and places for physical activity) with outcome (i.e., BMI percentile) adjusting for all covariates (i.e., neighborhood income, walkability, aesthetics, safety from crime and physical activity), as fixed effects and participant clustering in neighborhoods and school groups was adjusted-for as a random effect in the models. Mixed linear regression analyses are an extension of simple linear regression analyses to allow for both fixed and random effects [33]. By adjusting for physical activity, our models estimate the direct effect of the built environment on BMI, independent of individual movement levels. This strategy was chosen to control for the energy expenditure side of the energy balance equation, although we acknowledge that physical activity may also serve as a potential mediator in the causal chain. Before conducting the models, multicollinearity diagnostics were performed to ensure that our findings were not driven by redundant associations between perceived and objective measures. The Variance Inflation Factor for all predictors, including neighborhood SES and the environmental variables, was below 3.0. This indicates that the observed independent associations are statistically robust and that the models are not biased by SES-linked misclassification or high correlation between exposure variables. The t-Student test was used to compare continuous measurements, and the chi-square test was used to compare proportions (i.e., weight status categories) between male and female groups. Statistical significance was set at *p* = 0.05.

## 3. Results

Table 1 shows the means, standard deviations, and comparisons between boys and girls in the study variables. There was greater access to fresh food markets in the neighborhoods where the girls lived (*p* = 0.043). In addition, the perception of neighborhood safety from crime was higher among parents who had sons than among those who had daughters (*p* = 0.022). The level of physical activity was higher in boys than in girls (*p* < 0.001).

**Figure 2:**
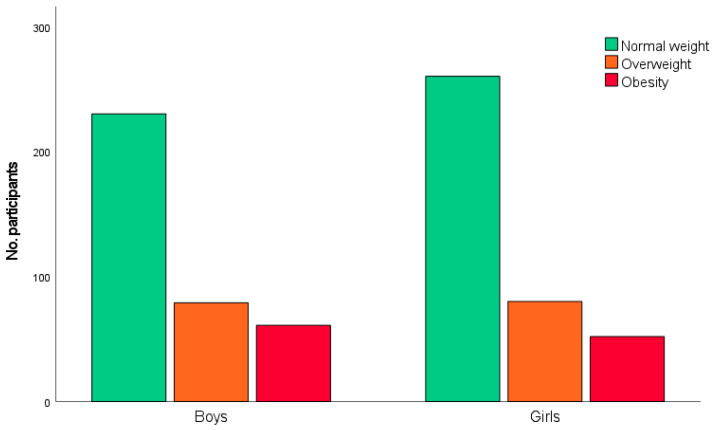
Distribution of weight categories between boys and girls.

Figure 2 shows the distribution by sex in the different body weight categories. The percentage of overweight boys and girls was 21.8% and 20.9%, respectively. Obesity was 16.8% in boys and 13.6% in girls. The chi-square test of independence revealed that the association between body weight category and sex was not statistically significant (χ^2^(2) = [1.93], *p* = 0.382).

Table 2 presents the associations, in the whole sample, between the independent variables and BMI percentile. Being a boy was positively associated with BMI percentile (*p* = 0.003), whereas physical activity was negatively related to this outcome (*p* = 0.028).

Table 3 shows the results of the mixed-model regression model stratified by sex. In the group of boys, none of the variables related to the accessibility to food and physical activity destinations were significantly associated with BMI percentile. However, access to healthy restaurants (*p* = 0.035) as well as neighborhood income (*p* = 0.049) were negatively associated with BMI percentile among girls.

## 4. Discussion

The present study analyzed how children’s spatial access to food destinations and places for physical activity, along with neighborhood walkability and SES, were related to BMI, from a sex-specific perspective. Our findings provide evidence that certain neighborhood characteristics (particularly access to healthy food destinations and neighborhood SES) are related to children’s body weight, with significant sex-specific differences. These results are consistent with previous evidence supporting the idea that a favorable built environment might protect against excess weight in children [34,35,36,37]. However, it is important to contextualize these findings within the European urban environment. Unlike the North American context, where ‘food deserts’ (areas with a total lack of healthy food access) are more prevalent, European cities typically feature higher residential/intersection density and mixed land use [38,39]. The relative absence of true ‘food deserts’ in our study area may attenuate the detectable associations between food accessibility and BMI, as most children have some degree of proximity to food outlets. Furthermore, this urban configuration often leads to a limited exposure contrast, as the ‘dose’ of environmental attributes may be uniformly high across the sample. Such a ‘ceiling effect’—where access to supermarkets, parks, or walkable streets is consistently high—reduces the statistical variability needed to detect meaningful effects on BMI. This lack of a stark contrast in accessibility, compared to more heterogeneous or sprawled regions, likely contributes to the modest or null associations observed for certain variables. Literature has shown that healthy food access, better walkability characteristics and access to physical activity facilities are associated with lower risks of childhood obesity. Nevertheless, not all previous studies report consistent associations in the aforementioned variables [34,36]. One reason for this inconsistency could be the lack of a standardization of measures across studies or the modifying effect of socio-demographic characteristics. In relation to socio-demographics, a recent systematic review of longitudinal studies reported that associations between neighborhood environment and child obesity vary by sex, age, and socio-cultural context [34]. This highlights that our results may not be directly generalizable to regions with more pronounced inequalities in food and physical activity infrastructure, such as highly suburbanized or car-dependent areas. Within the food environment domain, the strongest evidence of adverse impact was for unhealthy food environments (i.e., high density of fast-food restaurants), but this negative effect was only apparent among girls [34].

This pattern is in line with our findings, where significant environmental associations were observed only in female children. While the reasons for these sex-specific differences remain to be fully elucidated, we hypothesize several potential mechanisms. Girls may rely more frequently on the immediate neighborhood for daily food and activity, making perceived local accessibility a potentially stronger driver of their behaviors. Another hypothetical explanation could be related to differences in the timing of biological maturation [10]. According to authors such as Chen and Wang [10], girls typically enter puberty earlier than boys, a period during which weight gain may be more susceptible to environmental influences. Beyond biological factors, behavioral and social mediators may also play a role. For instance, parental control and safety concerns often restrict girls’ independent mobility more than in boys, potentially making girls more dependent on the specific amenities available in their immediate vicinity. Furthermore, social norms regarding body image and physical activity may lead girls to interact with their built environment differently, perhaps relying more on neighborhood aesthetics and safe walking paths, whereas boys’ activity levels may be driven by structured environments like school or organized sports. Future research should investigate these mediating pathways to confirm these hypotheses.

Moreover, physical activity was inversely associated with BMI in the overall sample, emphasizing the protective role of regular physical activity. However, in our study, access to physical activity facilities (i.e., public parks and, sports and recreational facilities) did not directly correlate with BMI, indicating that infrastructure alone may be insufficient without strategies to encourage use—especially tailored to children’s sex-specific needs.

Our findings suggest that public health and urban planning policies should adopt sex-sensitive approaches to address the distinct environmental drivers of childhood obesity. Improving neighborhood walkability or providing parks might not be enough; targeted zoning regulations would be needed. It is crucial to ensure equitable access to healthy food options and supportive neighborhood conditions, especially in areas with varying SES. Specifically, local governments could implement zoning incentives to attract full-service supermarkets to ‘food deserts’ or establish ‘healthy food zones’ around schools that limit the density of fast-food outlets. Additionally, the availability of safe, accessible, and appealing spaces for physical activity should be ensured. Interventions should recognize that boys and girls may respond differently to environmental factors and urban design. For instance, neighborhood-level improvements in healthy food access and socio-economic supports may particularly benefit girls; school- and sport-based interventions may be needed for boys. Nevertheless, given the modest and sex-specific associations observed in this study, these environmental policies should not be viewed as standalone solutions. To meaningfully impact childhood obesity, built environment interventions must be integrated into a broader, multi-level strategy that combines urban planning with family-, school-, and behavior-focused programs. Such a holistic approach would address both the structural barriers in the neighborhood and the individual lifestyle factors that collectively drive energy balance in children.

The strengths of this study include the integration of multiple environmental domains (food environment, physical activity destinations, walkability and SES) and a sex-sensitive approach. Limitations include the cross-sectional design, which precludes causal inference; reliance on some variables (i.e., parental perceptions) that may be subject to reporting bias; and limited generalizability beyond the studied area. Additionally, while our focus was on the built environment, this represents only one component of the complex causal web influencing childhood obesity. A key limitation was the lack of data on broader determinants of BMI, such as detailed dietary intake and eating patterns (e.g., fruit and vegetable consumption), screen time, and sleep duration, all of which are critical behavioral drivers of energy balance [40,41]. Furthermore, biological and familial factors were not fully accounted for, including parental BMI, family lifestyle, and the participants’ pubertal status or biological maturation, which may significantly influence weight gain during childhood and adolescence. Future research should adopt a more holistic approach by integrating these individual (e.g., dietary quality) and family-level variables with environmental attributes to better understand their synergistic effects on BMI. Specifically, there is a need for longitudinal designs that follow cohorts over time. Such approaches would allow for a more robust assessment of causality and help determine how changes in neighborhood characteristics—rather than just static exposure—directly influence the trajectory of children’s weight status throughout different developmental stages. Additionally, the inclusion of physical activity as a covariate means that our study measures direct effects. Future research using mediation analysis or structural equation modeling could further disentangle the total effect by quantifying the specific proportion of the environmental impact that is mediated through physical activity versus other lifestyle factors.

## 5. Conclusions

The relationship between built environmental attributes and BMI varies significantly between boys and girls. Understanding these differences is key for designing effective public health interventions with the aim of reducing childhood obesity. Future research should continue to explore these relationships to inform targeted interventions that consider sex-specific needs. Longitudinal research is needed to clarify causal pathways, explore behavioral mediators, and evaluate how environmental interventions may differentially impact boys and girls across diverse socio-economic contexts.

## Figures and Tables

**Table 1:** Descriptive data and comparisons between boys and girls.

	Boys (*n* = 363)		Girls (*n* = 383)			
	Mean	SD	Mean	SD	*t-student*	*p*
Convenience store	4.49	0.88	4.53	0.81	−0.59	0.279
Supermarket	4.42	0.84	4.36	0.88	0.94	0.174
Healthy restaurant proximity index	0.54	1.36	0.40	1.29	1.40	0.081
Coffee place	4.54	0.85	4.55	0.87	−0.24	0.405
Fresh food market	90.41	26.05	93.44	21.13	−1.72	**0.043**
Sports and recreational facilities	3.79	1.08	3.71	1.09	1.06	0.145
Public park	4.27	0.81	4.30	0.77	−0.60	0.274
Neighborhood income	12,711.36	3418.72	12,921.55	3569.09	−0.77	0.220
Neighborhood walkability	1.94	1.53	2.11	1.44	−1.48	0.070
Neighborhood aesthetics	2.53	0.81	2.59	0.79	−0.96	0.169
Neighborhood safety from crime	2.75	1.03	2.59	1.00	2.02	**0.022**
Physical activity	3.43	0.74	3.14	0.77	4.43	**<0.001**
BMI percentile (age and sex-adjusted)	67.58	27.86	65.22	27.91	1.15	0.124

Notes: SD: standard deviation; BMI: body mass index. Bold values indicate statistically significant differences (*p* < 0.05).

**Table 2:** Mixed-model regression results for relationship between independent variables and BMI percentile in whole sample.

Predictor	β	SE	*t*	*p*	95% CI
Gender					
Boys	8.16	2.77	2.95	**0.003**	2.72/13.59
Girls	ref.				
Convenience store	−1.19	2.18	−0.54	0.586	−5.46/3.09
Supermarket	−0.43	2.07	−0.21	0.835	−4.50/3.64
Healthy restaurant proximity index	−0.52	1.02	−0.51	0.612	−2.53/1.49
Coffee place	0.52	2.03	0.26	0.798	−3.46/4.50
Fresh food market	0.03	0.08	0.36	0.718	−0.13/0.19
Sports and recreational facilities	−1.48	1.40	−1.06	0.290	−4.24/1.27
Public park	0.44	1.97	0.22	0.823	−3.44/4.32
Neighborhood income	−0.00	0.00	−1.00	0.318	−0.00/0.00
Neighborhood walkability	−0.92	1.38	−0.67	0.506	−3.63/1.79
Neighborhood aesthetics	1.41	1.79	0.79	0.430	−2.10/4.92
Neighborhood safety from crime	−1.12	1.39	−0.81	0.420	−3.85/1.61
Physical activity	−4.04	1.84	−2.20	**0.028**	−7.65/−0.43
Age	−0.30	1.59	−0.19	0.852	−3.41/2.82

Notes: SE: standard error; CI: confidence interval; ref.: reference. Participant clustering in neighborhoods and school groups was adjusted for as a random effect in the models. Bold values indicate statistically significant differences (*p* < 0.05).

**Table 3:** Mixed-model regression results for relationship between independent variables and BMI percentile in boys and girls.

	Boys					Girls				
Predictor	β	SE	*t*	*p*	95% CI	β	SE	*t*	*p*	95% CI
Convenience store	0.12	2.92	0.04	0.966	−5.64/5.89	−3.34	3.22	−1.03	0.302	−9.69/3.02
Supermarket	−3.93	2.98	−1.32	0.190	−9.81/1.96	1.49	2.81	0.53	0.595	−4.04/7.02
Healthy restaurant proximity index	2.25	1.40	1.61	0.108	−0.50/5.01	−3.20	1.50	−2.13	**0.035**	−6.16/−0.23
Coffee place	0.64	3.06	0.21	0.834	−5.40/6.69	1.26	2.70	0.47	0.642	−4.07/6.59
Fresh food market	0.01	0.13	0.11	0.910	−0.24/0.27	0.04	0.11	0.39	0.695	−0.17/0.25
Sports and recreational facilities	−1.08	2.10	−0.52	0.606	−5.23/3.06	−1.78	1.92	−0.93	0.354	−5.55/2.00
Public park	−0.82	2.72	−0.30	0.764	−6.17/4.54	1.41	2.84	0.50	0.619	−4.18/7.01
Neighborhood income	0.00	0.00	0.69	0.489	0.00/0.00	−0.00	0.00	−1.98	**0.049**	−0.00/−0.00
Neighborhood walkability	−0.70	1.85	−0.38	0.704	−4.35/2.94	−0.78	1.95	−0.40	0.689	−4.61/3.06
Neighborhood aesthetics	2.04	2.47	0.82	0.411	−2.83/6.90	0.78	2.59	0.30	0.764	−4.33/5.89
Neighborhood safety from crime	−0.51	1.89	−0.27	0.789	−4.24/3.23	−1.55	2.01	−0.77	0.442	−5.51/2.42
Physical activity	−2.61	2.65	−0.98	0.326	−7.84/2.62	−4.42	2.52	−1.75	0.081	−9.38/0.55
Age	−2.36	2.29	−1.03	0.304	−6.88/2.16	1.75	2.21	0.79	0.430	−2.61/6.11

Notes: SE: standard error; CI: confidence interval. Participant clustering in neighborhoods and school groups was adjusted-for as a random effect in all models. Bold values indicate statistically significant differences (*p* < 0.05).

## Data Availability

The dataset supporting the conclusions of this article is not publicly available due to privacy or ethical restrictions but is available upon reasonable request to the corresponding author.

## Abbreviations

The following abbreviations are used in this manuscript:

BEACH	Built Environment and Active Children
BMI	Body Mass Index
CDC	Centers for Disease Control and Prevention
GHSCI	Global Healthy and Sustainable City Indicators
GIS	Geographic Information System
IPEN	International Physical Activity and the Environment Network
NEWS-Y-IPEN	Neighborhood Environment Walkability Scale for Youth—IPEN
OMS	OpenStreetMap
PAQ-C	Physical Activity Questionnaire for Children
SD	Standard deviation
SES	Socio-economic status
